# Nano-structured glaucoma drainage implant safely and significantly reduces intraocular pressure in rabbits via post-operative outflow modulation

**DOI:** 10.1038/s41598-020-69687-4

**Published:** 2020-07-31

**Authors:** Kunal S. Parikh, Aditya Josyula, Revaz Omiadze, Ju Young Ahn, Youlim Ha, Laura M. Ensign, Justin Hanes, Ian Pitha

**Affiliations:** 1 Center for Nanomedicine, Johns Hopkins University School of Medicine, Baltimore, MD 21231 USA; 2 Department of Biomedical Engineering, Johns Hopkins University School of Medicine, Baltimore, MD 21205 USA; 3 Center for Bioengineering Innovation & Design, Johns Hopkins University, Baltimore, MD 21218 USA; 4 Department of Ophthalmology, Wilmer Eye Institute, Johns Hopkins University School of Medicine, Baltimore, MD 21231 USA; 5 Department of Chemical and Biomolecular Engineering, Johns Hopkins University, Baltimore, MD 21218 USA; 6 Department of Pharmacology and Molecular Sciences, Johns Hopkins University School of Medicine, Baltimore, MD 21205 USA; 7 Glaucoma Center of Excellence, Wilmer Eye Institute, Johns Hopkins University, Baltimore, MD 21287 USA; 8 Departments of Environmental Health Sciences, Oncology, and Neurosurgery, Johns Hopkins University School of Medicine, Baltimore, MD 21231 USA

**Keywords:** Nanoscale materials, Health care, Nanoscience and technology, Engineering, Biomedical engineering, Materials science, Materials for devices

## Abstract

Glaucoma is a leading cause of irreversible vision loss predicted to affect more than 100 million people by 2040. Intraocular pressure (IOP) reduction prevents development of glaucoma and vision loss from glaucoma. Glaucoma surgeries reduce IOP by facilitating aqueous humor outflow through a vent fashioned from the wall of the eye (trabeculectomy) or a glaucoma drainage implant (GDI), but surgeries lose efficacy overtime, and the five-year failure rates for trabeculectomy and tube shunts are 25–45%. The majority of surgical failures occur due to fibrosis around the vent. Alternatively, surgical procedures can shunt aqueous humor too well, leading to hypotony. Electrospinning is an appealing manufacturing platform for GDIs, as it allows for incorporation of biocompatible polymers into nano- or micro-fibers that can be configured into devices of myriad combinations of dimensions and conformations. Here, small-lumen, nano-structured glaucoma shunts were manufactured with or without a degradable inner core designed to modulate aqueous humor outflow to provide immediate IOP reduction, prevent post-operative hypotony, and potentially offer significant, long-term IOP reduction. Nano-structured shunts were durable, leak-proof, and demonstrated biocompatibility and patency in rabbit eyes. Importantly, both designs prevented hypotony and significantly reduced IOP for 27 days in normotensive rabbits, demonstrating potential for clinical utility.

## Introduction

Glaucoma is a disease of the optic nerve and a leading worldwide cause of irreversible vision loss. Over 60 million people were affected by glaucoma in 2010, and more than 12 million will be bilaterally blind due to glaucoma by 2020. Glaucoma will affect more than 110 million individuals by 2040^1,2^. Intraocular pressure (IOP) reduction prevents glaucoma progression and vision loss^3,4^. Clinically, ophthalmologists use a variety of approaches to lower IOP, including medications, laser procedures, and/or incisional surgeries. Topical medications are first-line therapy in most cases as they reduce IOP while avoiding complications such as bleeding, infection, and hypotony that can reduce vision and are associated with incisional surgeries. However, topical medications do not always lower IOP sufficiently to halt disease progression, are associated with poor patient adherence, and can cause symptomatic eye surface irritation and redness^5^.

Earlier surgical intervention can remove the need for eye drops, the burden of adherence, and risks of topical medication, which could significantly reduce the economic burden of glaucoma. However, current gold standard surgical treatments for glaucoma are variable, lengthy, highly invasive, burdensome, and have high rates of complications and failure. Risks associated with IOP-reducing surgeries are partly attributed to a lack of procedural standardization^6,7^. The most frequently performed IOP-lowering surgeries create a vent that releases pressure from the anterior chamber of the eye to the subconjunctival space^8^. The specific IOP at which vents function is often determined by ad hoc interventions performed by the surgeon that are at risk for variability from surgeon to surgeon and by individual surgeons from procedure to procedure. The trabeculectomy, performed in 23,877 Medicare beneficiaries in 2012, is the most commonly performed incisional glaucoma surgery^9^. Fluid flow through the venting region of a trabeculectomy is regulated by the tension created by sutures that secure a flap of scleral tissue. This process leads to inconsistent outcomes, as sutures often need to be cut in the post-operative period due to inadequate IOP reduction, and the frequency of post-operative hypotony can exceed 10%^10,11^. Hypotony—a term that describes low IOPs that lead to vision problems—most commonly occurs at IOPs lower than 5 mmHg and can be associated with blurry vision, macular folds, choroidal detachments, and anterior chamber shallowing^12^. Aqueous tube shunts that drain to subconjunctival reservoirs offer an alternate surgical approach to IOP reduction that has increased in popularity over the past decade. Tube shunt insertion in Medicare beneficiaries increased from 6,307 in 2002 to 12,021 in 2012^9^. However, these devices are susceptible to post-operative complications, and surgeons have had to devise additional strategies to minimize post-operative hypotony. The Baerveldt glaucoma drainage implant (GDI) requires formation of a fibrotic capsule around the implant to prevent hypotony, but this process does not occur until several weeks following surgery. In order to avoid hypotony in the immediate post-operative period, the tube is occluded or tied off for the initial 4–6 post-operative weeks to allow capsule formation^13^. This strategy either allows for no IOP reduction in the immediate post-operative period or requires the surgeon to create venting incisions in the tube that can lead to inconsistent outcomes^14,15^. The Ahmed GDI contains a valve that is designed to vent above IOPs of 8 mmHg; however, 16% of valves did not meet this specification (either vented below 7 mmHg or above 15 mmHg) when tested prior to implantation^16^.

More recent evolutions of GDIs, minimally invasive glaucoma surgery (MIGS) devices, standardize venting IOP and reduce the risk of post-operative hypotony by utilizing dimensions modeled by the Hagen-Poiseuille equation (HPE):

1 $$\Delta P=\frac{8\mu LQ}{\pi {r}^{4}}$$

Equation 1 calculates resistance of an incompressible, Newtonian fluid in laminar flow through a cylindrical tube^17^. When applied to IOP reduction in the eye, the venting resistance (*ΔP*) to fluid outflow is inversely proportional to radius raised to the fourth power (*r*^*4*^) and directly proportional to tube length (*L*), aqueous humor volumetric flow rate (*Q*), and aqueous humor viscosity (*µ*). For example, the XEN 45 Gel Stent has an internal diameter of 45 µm and a length of 6 mm^17–19^. Fluid flowing through this device at a physiological flow rate of 180 µL h^−1^ creates a pressure differential of 7.6 mmHg, theoretically avoiding IOPs associated with the complications of hypotony^17^. The dimensions of the PreserFlo (inner luminal diameter of 70 µm, length of 8.5 mm) are similarly designed to maintain an IOP greater than 5 mmHg.^20,21^ However, in clinical practice, MIGS devices continue to suffer from complications due to fibrosis and hypotony, and due to their static design, do not enable optimal IOP reduction through all phases of the post-operative period^22,23^. Overall, these cases highlight the need for a more standardized, safer approach to glaucoma surgery that evolves in accordance with the biological response to surgery.

Here, we sought to develop a new GDI composed of materials that are biocompatible and non-immunogenic, that integrates into ocular tissue in order to minimize fibrosis, and modulates its outflow constraints in accordance with the wound healing process in order to both prevent hypotony and enable significant, long-term IOP reduction. Electrospinning is a promising platform for the development of GDIs for glaucoma surgery, as it allows for incorporation of almost any absorbable or non-absorbable polymer into nano- or micro-fibers which can be configured into devices of myriad dimensions and conformations^24,25^. Importantly, nanoscale features have been shown to mimic a more native environment for cells, down-regulate fibrosis, and limit protein adsorption, which has been previously implicated in glaucoma device fouling and failure^26,27^. To date, electrospinning has been used widely in tissue engineering, and more recently, in the manufacture of medical devices^28^. Here, we describe an electrospinning platform used to create small lumen cylindrical tubes with the potential to directly integrate into ocular tissue and modulate aqueous humor outflow over time, and explore whether these devices are suitable for use as GDIs. Our 1st generation embodiment composed of non-degradable polyethylene terephthalate (PET) nanofibers, PET shunt (PS), features a static 50 µm inner lumen in order to determine the viability of an electrospun GDI. Our 2nd generation embodiment, the Pressure Control Shunt (PCS), features a 75 µm inner lumen, a non-degradable outer core composed of PET, and a degradable inner core composed of polyglycolide (PGA) that absorbs over three weeks, thereby increasing the inner lumen diameter to 100 µm in order to safely achieve optimal IOP reduction. A control PS (CPS) with a static 100 µm diameter was also manufactured to evaluate the utility of post-operative outflow modulation via inner core degradation. Each embodiment was 6 mm in length and implanted in a standalone manner without a drainage plate.

## Results

### Simulated pressure change

**Figure 1:**
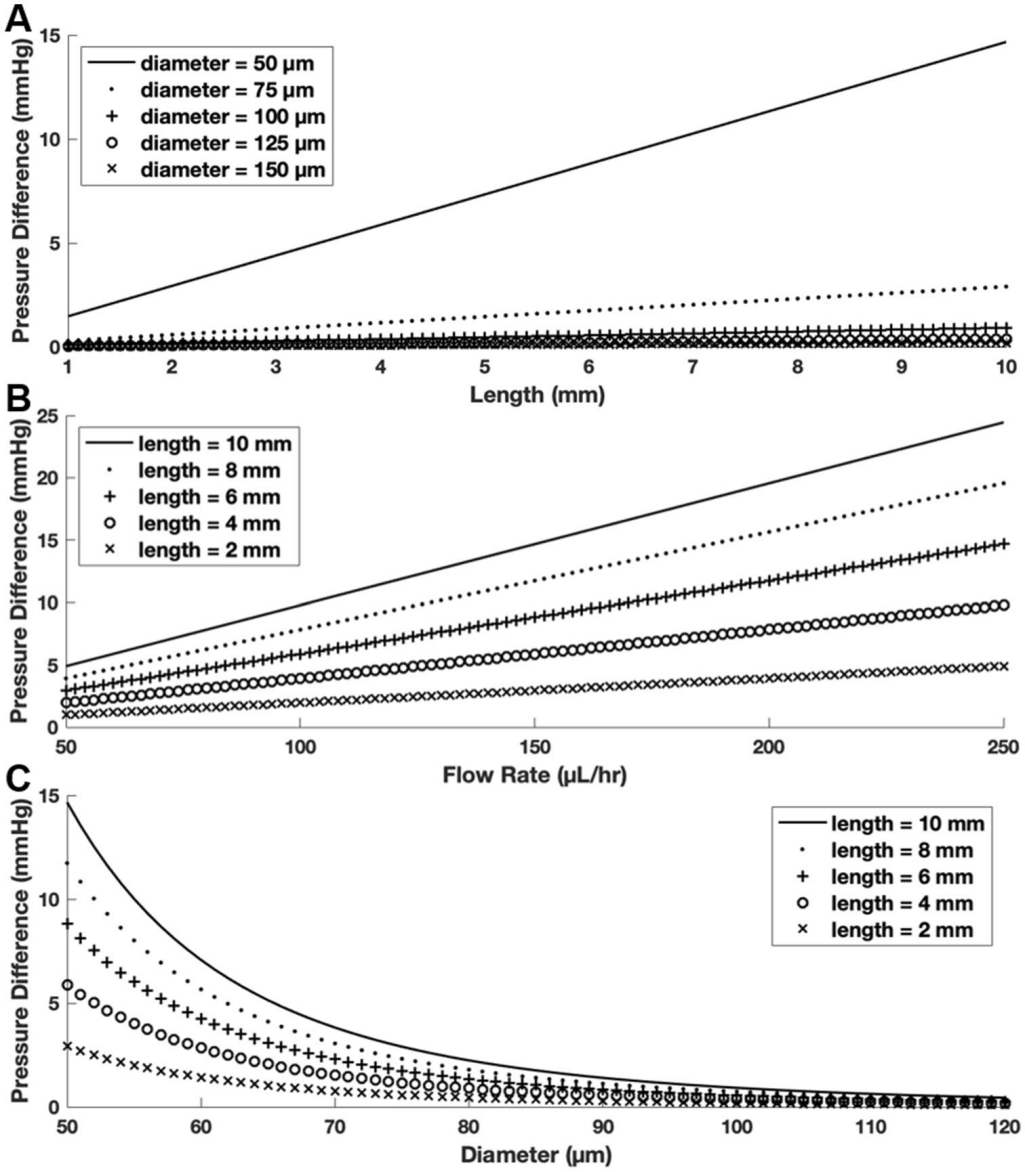
Model of pressure change through shunt. (**A**) Change in pressure difference with change in shunt length at a flow rate of 150 µL h^−1^. (**B**) Change in pressure difference with change in flow rate at a 50 µm shunt ID. (**C**) Change in pressure difference with change in diameter at a flow rate of 150 µL h^−1^.

Under physiological conditions, aqueous humor is an incompressible, Newtonian fluid^17^. Thus, by utilizing the HPE and physiological values of aqueous humor viscosity and flow rate, it is possible to determine the dimensions of a non-valved, cylindrical shunt capable of providing IOP reduction while also providing sufficient resistance to flow to prevent hypotony. Through variation of shunt diameter with flow rate held constant at 150 µL h^−1^, simulations revealed that a cylindrical shunt with inner diameter (ID) ≥ 100 µm will lead to hypotony at any physiologically relevant shunt length (Fig. 1A). However, a shunt with a 50 µm ID and greater than 4 mm in length may provide sufficient resistance to flow to maintain IOP above 5 mmHg. In order to achieve significant IOP reduction while simultaneously avoiding hypotony, 5–6 mm long shunts may be ideal. Through variation of shunt length with ID held constant at 50 µm, our simulation confirmed that a 6 mm long shunt is likely to avoid IOPs associated with hypotony, even if aqueous humor flow rate were to fall below its physiological average of 150 µL h^−1^ (Fig. 1B). Lastly, variation of shunt diameter from 50 to 150 µm in a 6 mm shunt demonstrates the broad range of pressures that can be achieved as the PCS inner core degrades and as ID increases (Fig. 1C). There is potential to dramatically reduce resistance to flow and modulate the IOP-lowering capacity of a shunt post-operatively through an expanding lumen.

### Nano-structured shunt characterization

**Figure 2:**
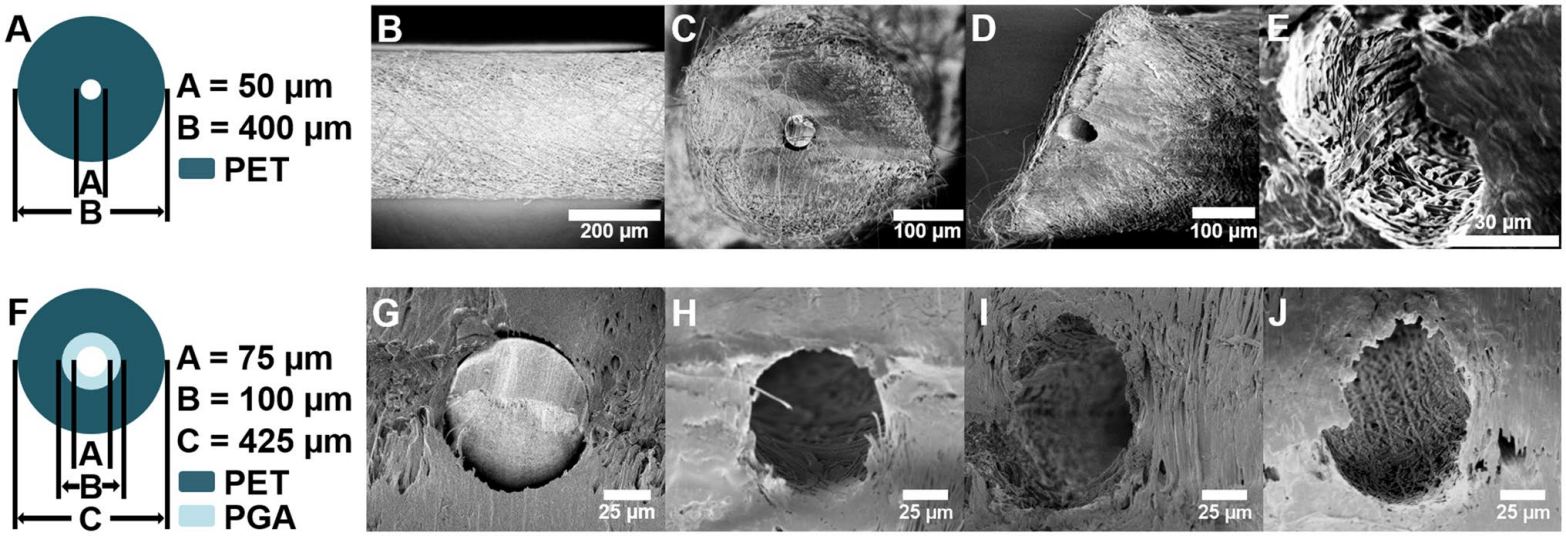
Nano-structured shunt design and characterization. (**A**) Design of PS. Inner lumen size is dictated by the diameter of the template wire. (**B**) Exterior of PS composed of nanofibers and revealing minimal porosity. (**C**) Cross-section of closed-lumen PS with 50 µm diameter template wire and (**D**) cross-section without template wire. (**E**) Cross-section of open-lumen PS following one week of continuous fluid flow demonstrating no signs of degradation or change in diameter. (**F**) Design of PCS. (**G**) Cross-section of PCS with 75 µm diameter template wire and (H) cross-section without template wire. (**I**) Cross-section of PCS after 14 days and (**J**) 28 days of in vitro fluid flow.

1st generation PS were designed with a static 50 µm ID (Fig. 2A) based on the results of modeling studies in order to prevent hypotony and determine the viability of an electrospun GDI composed of nanofibers. Nano-structured shunts were manufactured via an electrospinning-based platform in which voltage was applied to a viscous, volatile polymer solution flowing perpendicular to a rotating, grounded, stainless steel wire. This provided for a dense and uniform coating of polymeric nanofibers onto the template wire with low wall porosity following heat treatment (Fig. 2B). In order to achieve high strength and maintain lumen integrity in vivo, PS were manufactured with a wall thickness of 354 ± 15 µm, and composed of nanofibers, which have been shown to demonstrate a higher tensile modulus than larger diameter electrospun fibers^29^. 5, 6, and 7 mm long PS were manufactured with an internal lumen diameter of 50 µm formed after removal of the 50 µm template wire. Prior to in vitro flow studies, the shunt was cut to the appropriate size, and the template wire (Fig. 2C) was removed from the inner lumen (Fig. 2D). As shown in Fig. 2E, the lumen remained patent and maintained its size after one week of phosphate-buffered saline (PBS) flow in vitro. The inner surface of the shunt was composed of aligned nanofibers showing no degradation or breakage.

Following validation of PS, 2nd generation PCS were designed with a 75 µm ID, a 25 µm degradable inner core composed of PGA, and a non-degradable outer core composed of PET nanofibers (Fig. 2F) in order to provide immediate IOP reduction post-operatively while minimizing the risks of early, post-operative hypotony. Inner lumen expansion over time could additionally provide more significant IOP reduction following wound healing and reduce the likelihood of lumen obstruction. Scanning electron microscope (SEM) images of PCS cross-sections demonstrated integration of the inner and outer cores (Fig. 2G), an average outer diameter (OD) of 421 ± 8 µm, and an increase in ID from 77.7 ± 1 µm following manufacture (Fig. 2H) to 85.8 ± 1 µm, 87.6 ± 2 µm and 98.8 ± 2 µm following 7, 14 (Fig. 2I) and 28 days (Fig. 2J) of in vitro PBS flow, respectively.

### In vitro performance and flow through shunt

**Figure 3:**
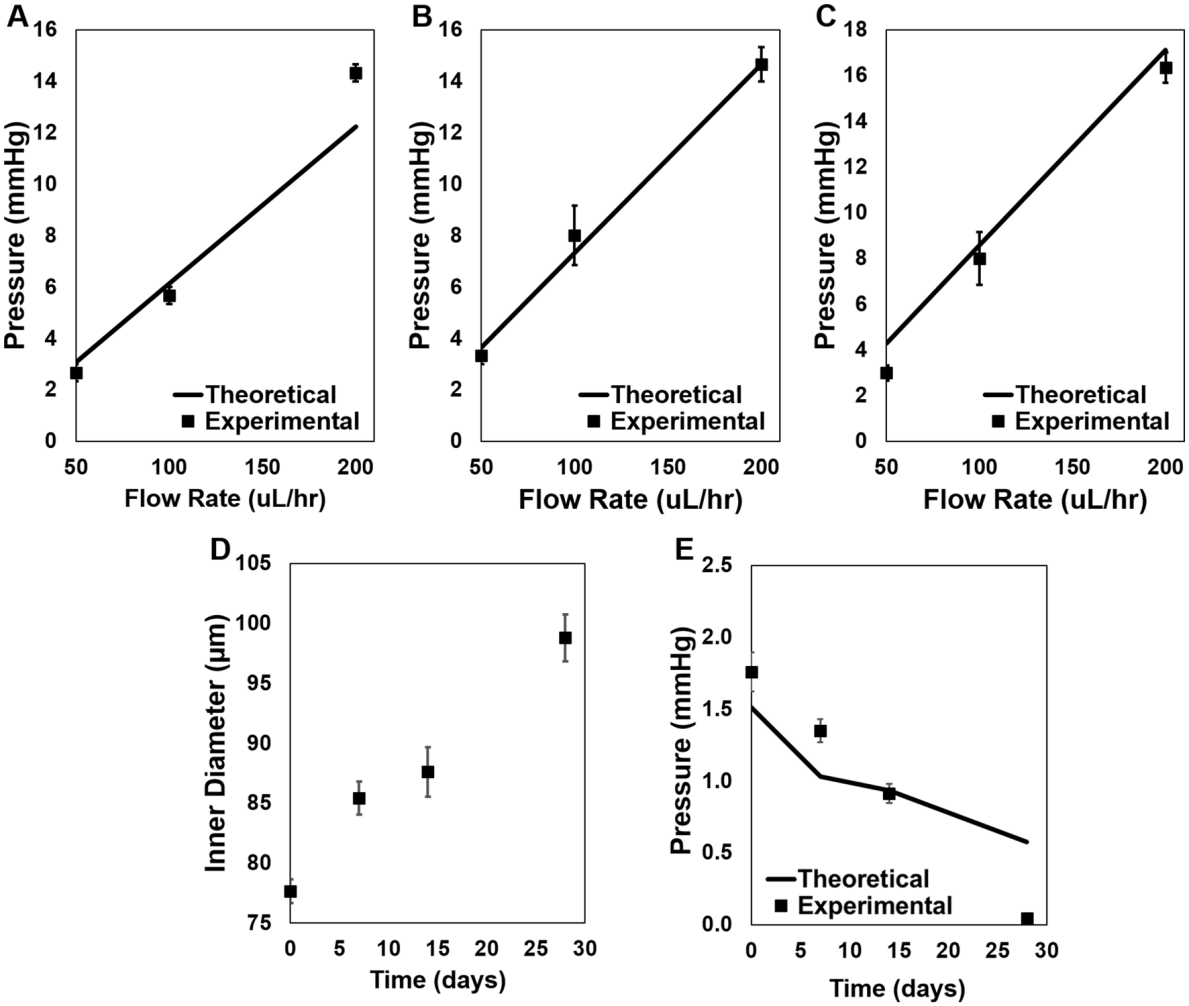
Theoretical and experimental pressure differential with flow of PBS through shunts. PS had a 50 µm ID and were (**A**) 5 mm, (**B**) 6 mm, or (**C**) 7 mm in length. PBS was pumped at 150 µL h^−1^ through 6 mm long PCS, resulting in increasing ID (**D**) and reduced resistance to flow over time (**E**).

PS and PCS were evaluated via in vitro fluid flow to determine if they are leak-proof, durable, and provide the resistance to flow predicted by the HPE. PBS was pumped through 5, 6, and 7 mm long PS at flow rates of 50, 100, and 200 µL h^−1^. Resistance to flow increased with faster flow rates and increased shunt length (Fig. 3A–C). At each shunt length, there was a strong correlation between the experimental measurements and theoretical pressure values predicted by the HPE. This correlation became stronger as shunt length surpassed 5 mm, and therefore, as entrance length increased. The percent difference between the slope of the linear trendline of experimental values and slope of the theoretical values was 29.3%, 1.4%, and 2.9%, respectively, for 5, 6, and 7 mm long shunts. Notably, PS maintained their shape (Fig. 2E) and did not leak throughout the duration of the study. PBS was then pumped continuously through 6 mm long PCS at 150 µL h^−1^ for 28 days with ID and pressure differential measured at 7, 14, and 28 days. The inner PGA core degraded fully over 28 days, with the ID ultimately reaching 98.8 ± 2 µm (Fig. 3D). Importantly, there was minimal variation in ID over the flow period, as the standard error remained ≤  ± 2 µm at all time points. No leakage was observed during the study and the resistance to flow decreased linearly throughout the study, leading to minimal resistance to flow at day 28 (Fig. 3E).

### IOP reduction in normotensive rabbits

6 mm long PS with either an open- or closed- (template wire not removed) lumen, PCS with an open lumen, and CPS with an open lumen were implanted in normotensive New Zealand White (NZW) rabbit eyes via an *ab externo* procedure designed to vent aqueous humor via subconjunctival outflow. Procedure time ranged from 8 to 12 min for all types of shunts. Open-lumen PS were implanted and evaluated in rabbit eyes in order to determine the viability of the nano-structured shunt platform in vivo*.* Closed-lumen PS provided a procedural control to determine whether the implantation procedure or the drainage implant contributed to IOP reduction. PCS were implanted to evaluate the potential clinical utility of a nano-structured shunt featuring a lumen that slowly expands from 75 to 100 µm. CPS with a 100 µm ID allowed for immediate evaluation of the biological response to a static-lumen shunt with the final diameter of PCS. Mean baseline and post-operative IOP measurements are listed in Table S1.

**Figure 4:**
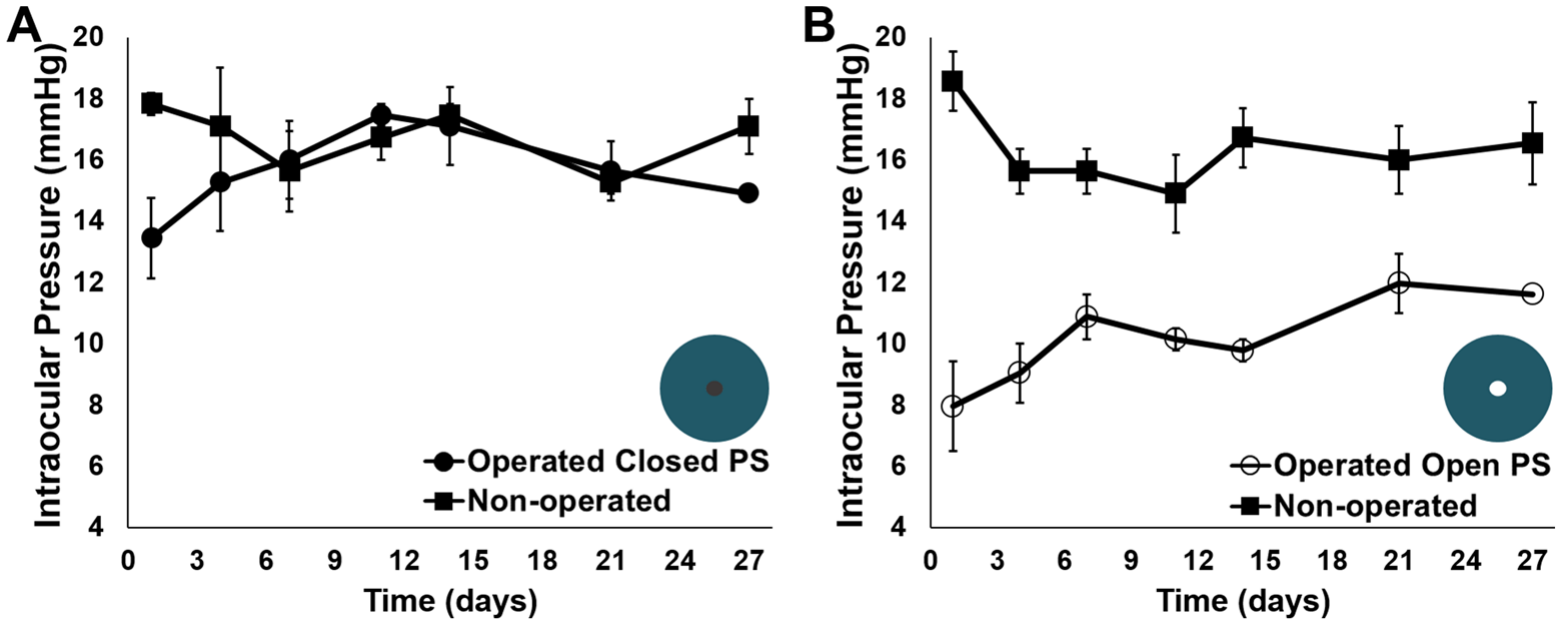
IOP measurements in rabbits following implantation of open- and closed-lumen PS. IOP was measured in both the operated and the healthy, non-operated contralateral control eyes of rabbits receiving either an open- or closed-lumen PS (open PS or closed PS in the figure caption). (**A**) Rabbit eyes implanted with a closed-lumen PS demonstrated no significant change in IOP in comparison to the contralateral control eye (p = 0.06). (**B**) Eyes implanted with an open-lumen PS demonstrated a significant reduction in IOP in comparison to the non-operated contralateral control eye (p < 0.0001) and to eyes containing closed-lumen PS (p < 0.0001).

The lowest average IOP for rabbits with either open-lumen (8.0 ± 1 mmHg) or closed-lumen PS (13 ± 1 mmHg) was observed 24 h post-operatively, demonstrating that the procedure itself may have contributed to IOP reduction in the immediate post-operative period (Fig. 4A,B). However, the IOP of operated rabbit eyes containing closed-lumen PS was not significantly different than that of the respective healthy, contralateral control eyes (p = 0.06), with an average IOP of 16 ± 1 and 17 ± 1 mmHg, respectively, indicating that the surgical procedure alone does cause IOP reduction. Hypotony and/or flattening of the anterior chamber were not observed in the immediate, early, intermediate, or late post-operative period. The reduction in IOP for eyes containing closed-lumen PS ranged from − 4 to 25% on individual days following implantation, with the most significant reduction observed immediately post-operatively. Operated rabbit eyes containing open-lumen PS demonstrated a statistically significant decrease in IOP in comparison to the control contralateral eye (p < 0.0001), with an average IOP of 11 ± 1 and 16 ± 1 mmHg throughout the study, respectively. IOP reduction ranged from 24 to 57% on individual days following implantation. The IOP of operated eyes receiving open-lumen PS was also significantly lower than that of operated eyes receiving closed-lumen PS (p < 0.0001), with an average IOP of 11 ± 1 and 16 ± 1 mmHg, respectively. Collectively, these results demonstrate that small-lumen shunts composed of PET nanofibers enable significant aqueous humor outflow in vivo without complications such as hypotony, erosion, shunt migration, or failure-inducing fibrosis.

**Figure 5:**
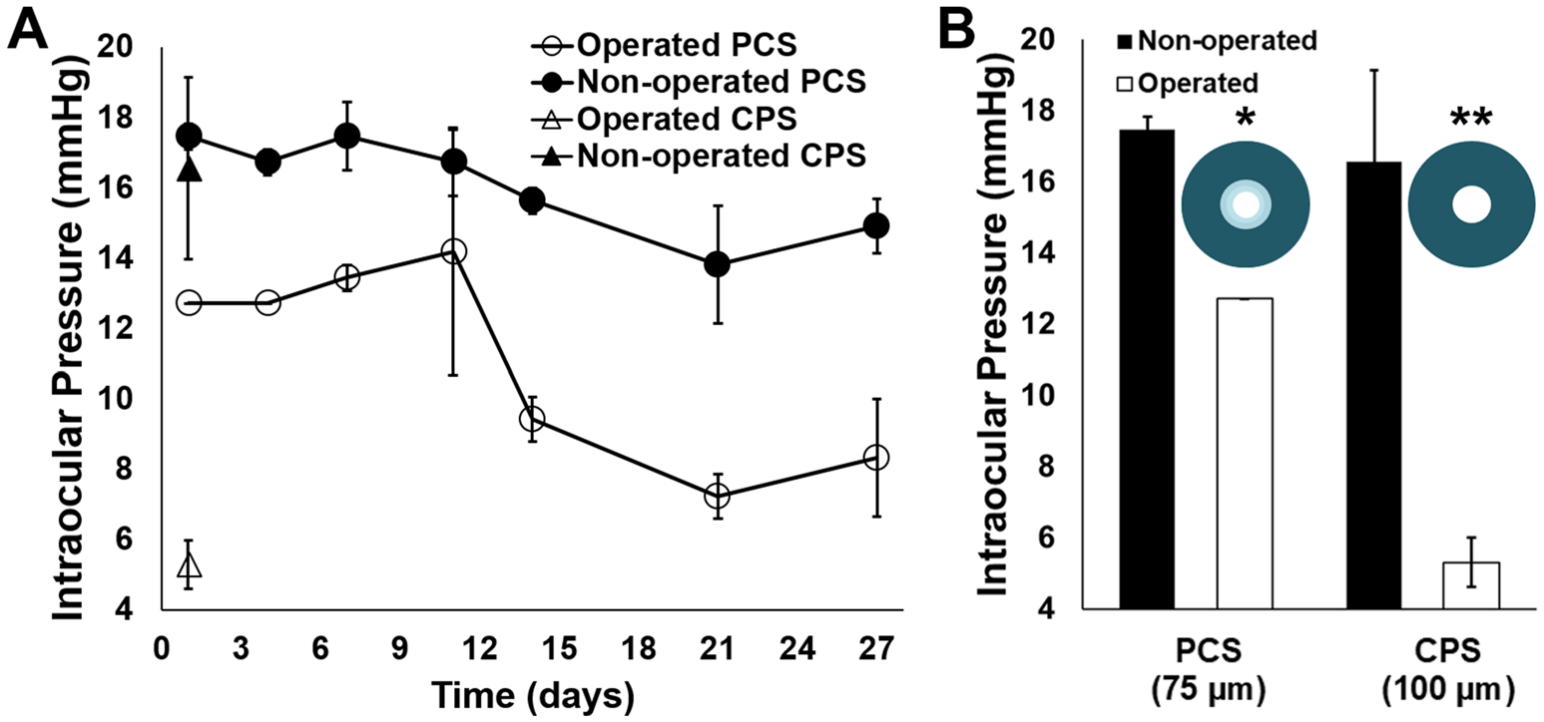
IOP measurements in rabbits following implantation of PCS and CPS. IOP was measured in both the operated and the healthy, non-operated, contralateral control eyes of rabbits receiving either an open-lumen PCS or CPS. (**A**) Eyes containing PCS demonstrated significant reduction in IOP in comparison to contralateral control eyes (p < 0.0001) and lowered IOP in accordance with inner core degradation without causing hypotony. (**B**) Eyes containing CPS demonstrated hypotony at post-operative day 1, and had significantly lower IOP in comparison to non-operated eyes and eyes with PCS. Difference in number of * indicates statistical significance at p < 0.01.

Following proof-of-concept of the nano-structured shunt platform, PCS and CPS were evaluated and compared in vivo in order to determine the viability of a degradable inner core that allows for implantation of shunts with a larger ID. PCS ID increased from 77.7 ± 1 µm at the time of implantation to 102 ± 3 µm after 27 days in rabbit eyes. In contrast, CPS were designed with a static 100 µm ID, which was theorized to immediately lead to hypotony post-operatively based on modeling in Fig. 1A. Hypotony (IOP readings ≤ 5 mmHg) was observed in 75% of rabbit eyes receiving CPS (Fig. 5A), and shallow anterior chambers were observed in 100% of these eyes at post-operative day 1. However, no rabbit eyes receiving PCS demonstrated hypotony or flattening of the anterior chamber throughout the post-operative period (Fig. 5B). PCS placement resulted in blebs that increased in extent and height over 10 days after implantation (Fig. S1). The smaller initial ID of PCS provided sufficient resistance to outflow in the first post-operative week to prevent hypotony while allowing for 23–27% IOP reduction in comparison to the contralateral non-operated eyes. At day 14, a sharp drop in IOP was observed in eyes containing PCS, correlating with the in vitro degradation profile of the inner core composed of PGA nanofibers. This reduction in IOP due to degradation of the inner core was sustained through the remainder of the study, ultimately leading to 6.6 mmHg of IOP reduction (44% reduction) on day 27 in comparison to the non-operated control. Operated rabbit eyes containing PCS demonstrated a statistically significant reduction in IOP in comparison to the control contralateral eye through the duration of the study (p < 0.0001), with an average IOP of 11 ± 3 and 16 ± 1 mmHg, respectively. 100% of PCS maintained patency during the degradation period and throughout the duration of the study, as indicated by fluorescein irrigation of the anterior chamber (Fig. S2).

### In vivo biocompatibility

**Figure 6:**
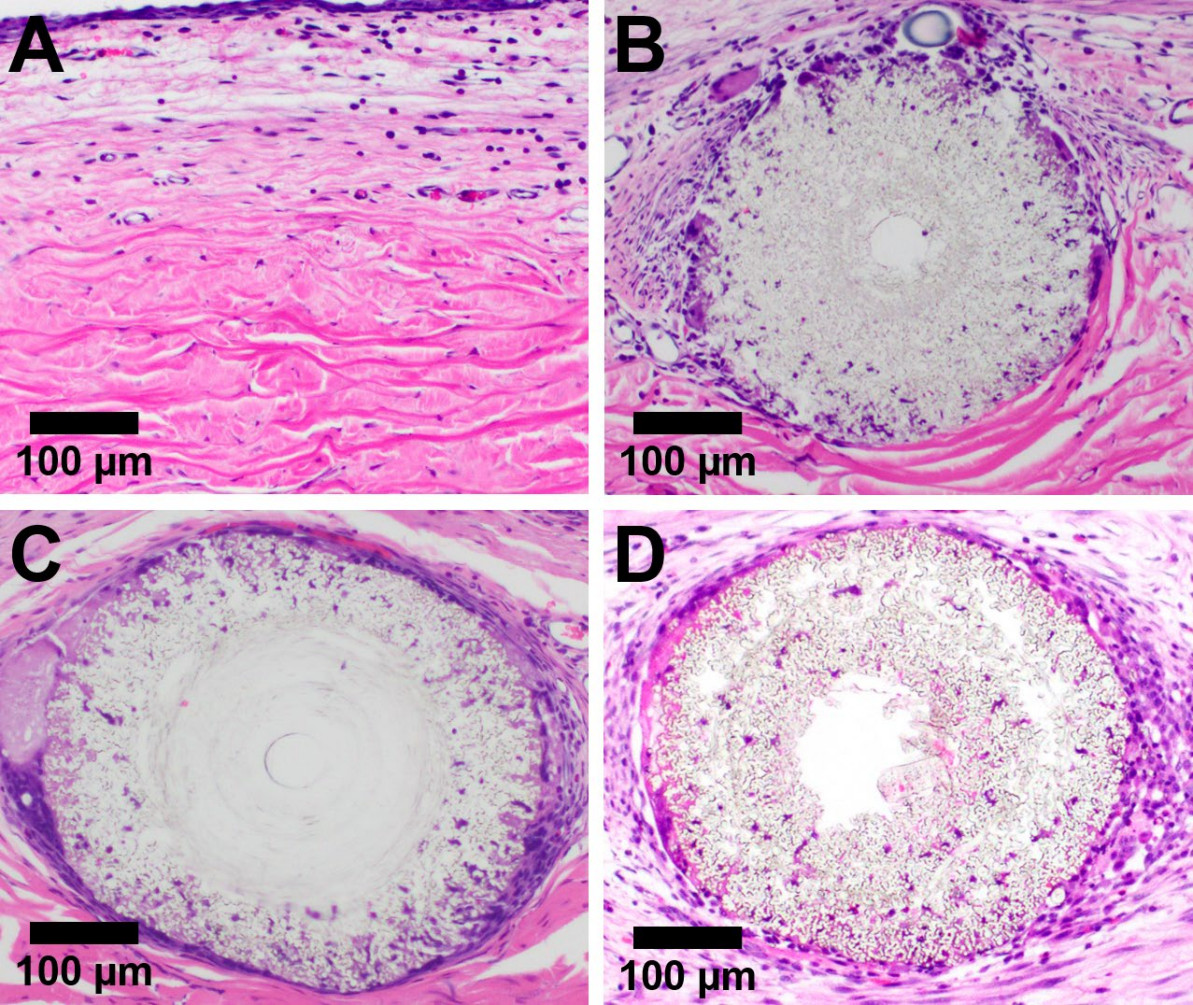
Glaucoma shunt biocompatibility. Representative images of (**A**) untreated subconjunctival rabbit tissue, (**B**) tissue surrounding an implanted, open-lumen PS, (**C**) tissue surrounding an implanted, closed-lumen PS, and (**D**) tissue surrounding an implanted, open-lumen PCS following hematoxylin and eosin (H&E) staining. The template wire became dislodged from the closed-lumen PS during sectioning.

All nano-structured shunts were well-tolerated by NZW rabbit eyes. Implanted shunts did not migrate into the anterior chamber during the study, and there were no cases of infection, hyphema, or inflammation (anterior segment inflammatory cells or fibrin) on exam under an operating microscope. Histological analysis of control subconjunctival tissue and tissue surrounding implanted shunts revealed the migration of cells to the periphery, and into the wall of both PS and PCS (Fig. 6A–D). The inner lumen remained clear of cells and tissue in all cases, correlating with observed in vivo patency. Formation of a mild fibrotic capsule was observed surrounding both PS and PCS. Neovascularization was not observed in the immediate vicinity of either open-lumen shunt. Notably, tissue sections also reveal that open-lumen PS maintain their original ID and OD, and lumen shape for at least 27 days following in vivo implantation. In contrast, PCS tissue sections reveal a larger, non-cylindrical lumen following inner core degradation. PCS dimensions, biocompatibility, and integration into the tissue are also confirmed via Masson’s trichrome staining (Fig. S3).

## Discussion

There is a need for glaucoma drainage devices with reduced post-operative complications, decreased reliance on ad hoc surgical “fixes,” and improved long-term IOP reduction outcomes. Here, we describe a novel, versatile platform for manufacture of nano-structured glaucoma drainage devices and evaluate multiple embodiments with dimensions derived from the HPE to preclude post-operative hypotony while enabling significant, long-term pressure reduction. Overall, nano-structured shunts demonstrated in vitro fluid flow consistent with the HPE, were leak-proof, and provided the appropriate strength, flexibility, and durability for successful implantation in rabbit eyes. Both PS and PCS demonstrated biocompatibility in rabbit eyes and were able to remain patent and significantly reduce IOP for at least 27 days in a normotensive rabbit model without hypotony.

To our knowledge, this is the first study to utilize an electrospun glaucoma drainage implant^30,31^. Electrospinning is a simple and versatile platform for the manufacture of medical devices. It is compatible with almost any natural or synthetic polymer, can incorporate prophylactic or therapeutic moieties, and be realized into a wide range of different morphologies or conformations^24,32^. Devices manufactured via electrospinning are also highly modifiable through coatings, laser cutting, and other processing modifications to tune almost every aspect of the device, including size, shape, strength, rigidity, porosity, degradability, glidability, and biocompatibility. For example, nanofibers have been shown to demonstrate increased strength and decreased porosity in comparison to microfibers^29,33^. In this study, the use of nanofibers to construct glaucoma shunts likely contributed to maintenance of an intact shunt lumen *in* vivo and prevention of leaking during flow studies in vitro*.* Heat treatment can be used to further increase shunt strength by aligning polymer chains and enhancing crystallinity, and if necessary, spray time can be modified to change wall thickness and modulate shunt flexibility^34^.

Electrospinning and its versatility are especially pertinent to the treatment of glaucoma with drainage implants, in which the use of slightly inflammatory materials such as polypropylene can lead to device failure, and where scar formation and fibrosis lead to significant complications and surgical failure^35,36^. PET was chosen in this application because it is generally regarded as safe (GRAS), has excellent biostability, a well-understood fibrotic response, and has been utilized in medical devices for over 50 years^37^. Similarly, PGA was chosen as it is a GRAS material with a well-characterized degradation profile that has been applied broadly in medical devices such as sutures^38^. However, should PET and PGA have not provided the appropriate strength, flexibility, or biological response, electrospinning permits facile interchange of polymer and/or modification of the manufacturing process to tailor the shunt to fit the clinical need. Moreover, the capacity of electrospinning to manufacture nanofibers and incorporate them into a medical device, is a substantial advantage in glaucoma treatment. In their natural environment, cells interact with nanoscale architecture and cues within the surrounding extracellular matrix. These cues can affect cell migration, morphology, orientation, and even gene regulation. Kam and coworkers demonstrated that nanoscale features with high aspect ratios decrease expression of several growth factors associated with fibrosis^26^. Their experiments revealed that these features can also reduce protein adsorption, which has been implicated in glaucoma device fouling and can lead to infection, increase in IOP, or device failure^27^. The methods used to manufacture marketed drainage devices are limited in their capacity to achieve these effects through manufacturing alone^39^. However, it is important to note that the porosity of nanofibrous devices may make them vulnerable to cell and/or tissue ingrowth, and their long-term use as permanent medical device implants is still under investigation^37,40^.

While electrospinning may be a suitable platform for manufacture of glaucoma shunts, the long-term safety and efficacy of shunts designed using the HPE is still under investigation in humans. The XEN Gel Stent was also designed using the HPE, but is vulnerable to hypotony and anterior chamber shallowing in the immediate post-operative period at ID dimensions of 45, 63 or 140 µm^18,41^. In a 12-month study of the XEN 45 Gel Stent in refractory glaucoma patients, 24.6% of patients experienced hypotony and 21.5% of patients required at least one bleb-needling procedure^41^. The small lumen of the XEN 45 Gel Stent and other MIGS devices likely reduces occurrence of persistent hypotony,however, it also limits the IOP reduction potential and may make device function more vulnerable to fouling over the long-term or blockage with fibrotic tissue or cellular debris^42^. Venting resistance is inversely proportional to *r*^4^, thus, small variations in shunt diameter can have a considerable effect on venting IOP. Additionally, small diameter shunts could potentially be obstructed with small amounts of tissue leading to complete device failure more commonly than shunts with larger diameters. Moreover, the HPE may not be able to accurately predict the performance of shunts in vivo, as shunts are curved to suit ocular anatomy and are affected by the surgical procedure and post-operative biological response which likely includes alternations in physiologic aqueous outflow pathways and changes in aqueous humor viscosity. In these studies, although HPE modeling predicted that a shunt with a 75 µm ID would cause hypotony, this was not observed in in vivo studies with PCS and immediate post-operative IOP reduction was not significantly more than seen with the 50 µm ID PS. These results highlight our incomplete understanding of the factors that influence IOP when these shunts are used in vivo versus in situ and in vitro models. Additionally, in vitro fluid flow studies demonstrated that shorter PS demonstrated a weaker correlation with the HPE. This may be due to the entrance length required to transition into stable, laminar flow, and is another factor to consider in the HPE-driven design of glaucoma shunts^43^.

Although the long-term efficacy of HPE-designed MIGS in humans is still under investigation, the performance of nano-structured shunts in healthy, normotensive rabbits in this study is promising. Without the use of mitomycin c (MMC), open-lumen PS shunts maintained their ID and significantly decreased IOP for at least 27 days in comparison to the non-operated contralateral eye. This is in contrast to previously conducted trabeculectomy and drainage implant procedures in healthy, normotensive NZW rabbits. Trabeculectomy procedures in these rabbits have been shown to fail within 7 days without the use of an antifibrotic or antimetabolic agent^44^. In a prospective, non-randomized study of fourteen patients, the MicroShunt demonstrated a 49.8% reduction in IOP in conjunction with MMC after 3 years; however, prior studies in NZW rabbits without MMC demonstrated no significant change in IOP in comparison to non-operated eyes or to implantation of silicone tubing with an ID of 300 µm^20,45^. This suggests that open-lumen PS may be able to vent significantly more aqueous humor in comparison to a healthy drainage system alone. The versatility of the nano-structured shunt platform also has potential to overcome major limitations of GDIs. Through use of a degradable core that allows modulation of aqueous humor outflow over time as the ID increases, PCS were able to prevent hypotony and reduce IOP by 54% in comparison to pre-operative IOP. Although it is important to note that weekly fluorescein irrigation of the anterior chamber to evaluate shunt patency may positively affect shunt function and capsule formation in these studies. A GDI capable of providing the safety profile of a MIGS device through a simple, reproducible insertion method, while achieving IOP reduction profiles similar to trabeculectomy would provide a compelling option for glaucoma patients requiring more significant pressure reduction than existing MIGS devices are able to deliver^46^. Additional longer-term studies and studies in diseased animal models may help to better understand these results, and to evaluate the potential clinical utility of this nano-structured shunt platform.

## Conclusion

This study demonstrated the manufacture of minimally invasive glaucoma drainage implants via electrospinning, and their evaluation in vitro and in vivo. PS and PCS were designed through simulation of the HPE under physiological conditions to promote long-term IOP reduction while preventing hypotony. In vitro studies revealed that nanofiber-based shunts were leak-proof and durable, and that fluid flow through the shunts behaves according to the HPE. In vivo experiments revealed that both PS and PCS are biocompatible and directly integrate surrounding issue and cells through their nanoarchitecture. Importantly, both embodiments provided significant IOP reduction while avoiding hypotony, with PS and PCS realizing a 30% and 44% reduction in IOP 27 days after implantation, respectively. This versatile fabrication platform could be used to manufacture next generation glaucoma shunts composed of almost any material and capable of additional functionality, including drug delivery.

## Methods

### Simulation of fluid flow through shunt

Modeling of pressure differential through the shunt was accomplished using the HPE equation in MATLAB (Mathworks, Natick, MA) by varying flow rate, shunt diameter, and shunt length. The viscosity (0.72 cP) and average flow rate (150 µL h^-1^) of human aqueous humor at physiological conditions were input for the values of *µ* and *Q*, respectively^17^.

### Electrospun shunt manufacture

Electrospun shunts were manufactured utilizing a grounded collector consisting of a drill chuck (McMaster-Carr, Elmhurst, IL) and parallel collector stand positioned perpendicular to the polymer jet. Stainless steel wire (McMaster-Carr) with a diameter of 50 (PS), 75 (PCS), or 100 µm (control PS (CPS)) was inserted through a 25 G, 3.81 cm long, blunt tip needle (Nordson EFD, Westlake, OH) which was then placed into the head of the drill chuck. PET (Nanofiber Solutions, Columbus, OH) was dissolved in 1,1,1,3,3,3-hexafluoro-2-propanol (HFIP; Sigma Aldrich, St. Louis, MO) at 10 wt% by stirring at 45 °C for 24 h. For PS, the polymer solution was then electrospun onto a 50 µm wire rotating clockwise at 175 rpm by applying 15 kV of voltage from a power supply (Glassman High Voltage, High Bridge, NJ) to a 20 G blunt tip needle (Nordson EFD) with the polymer solution flowing at a controlled rate of 1.5 mL h^−1^ using a syringe pump (New Era Pump Systems, Farmingdale, NY) until a 400 µm outer diameter (OD) was achieved. The CPS was manufactured similarly using a 100 µm template wire and an OD of 425 µm. For PCS, PGA was first dissolved in HFIP at 10 wt% prior to electrospinning at a flow rate of 850 µL h^−1^ at a voltage of 15 kV onto a rotating 75 µm template wire, until reaching an OD of 100 µm. PET was then spun around the PGA core until reaching an OD of 425 µm, as described above. All shunts were heated for 24 h at 100 °C prior to cooling to RT.

A total of 4 different shunt designs were manufactured for implanted. All implants were 6 mm in length, but varied in internal lumen diameter and design:

1. Open-lumen PET shunt (open PS) was fabricated from PET nanofibers with an internal lumen diameter of 50 µm.
2. Closed-lumen PET shunt (closed PS) has the same design and dimensions as the open PS but the internal lumen was occluded with a metal template wire
3. Pressure Control Shunt (PCS) was fabricated with an internal layer of PGA nanofibers surrounded by an external layer of PET nanofibers. The internal lumen expanded from 75 µm to 100 µm as the PGA degraded.
4. Control PS (CPS) shunt was fabricated from PET nanofibers with an internal lumen diameter of 100 µm.

### Shunt characterization

Shunt OD was measured via optical microscopy using an Eclipse TS100 (Nikon Instruments, Melville, NY). Inner lumen diameter and shunt morphology were examined via SEM at 1 kV with a LEO Field Emission SEM (Zeiss, Oberkochen, Germany) following desiccation for at least 24 h and sputter coating with 10 nm of Au/Pd. Imaging was conducted prior to removal of the template wire, after removal of the wire, and following in vitro fluid flow studies. Image analysis and shunt dimensions were measured using ImageJ (U. S. National Institutes of Health, Bethesda, MD, https://imagej.nih.gov/ij/).

### Evaluation of In vitro fluid flow through shunt

Experimental PS were cut to 5, 6, or 7 mm in length (n = 3 for each condition), after which the template wire was removed. The shunt was then connected in circuit to a syringe pump and manometer (NETECH, Farmingdale, NY) via the 25 G needle. Flow studies were conducted at RT. Dulbecco’s Phosphate Buffered Saline (PBS, ATCC, Manassas, VA) was pumped through the shunt at 50, 100, and 200 µL h^−1^. Prior to flow studies, PBS was pumped through a 25 G needle at the specified flow rate and the resulting pressure measurement was subtracted from the pressure observed with attachment of the shunt. Pressure measurements were recorded at least 30 min after a change in flow rate and only if the pressure remained constant for more than 5 min. Theoretical pressure estimates were obtained from the HPE with 25 °C PBS viscosity of 0.9 cP^47^. PCS (n = 3) were cut to 6 mm in length and evaluated similarly. Long-term flow studies were conducted at 37 °C by flowing PBS through PCS at 150 µL h^−1^ for 28 days.

### In vivo performance and iop measurement

All animals in these studies were cared for in accordance with protocols approved by the Johns Hopkins University Animal Care and Use Committee, with the ARVO Statement for the Use of Animals in Ophthalmic and Vision Research, and with the National Institutes of Health guide for the care and use of laboratory animals. Adult NZW rabbits (Robinson Services, Mocksville, NC) were sedated by subcutaneous injection of ketamine (75 mg kg^−1^, Fort Dodge Animal Health, Fort Dodge, IA) with xylazine (5 mg kg^−1^, VedCo Inc., Saint Joseph, MO). A drop of 0.5% proparacaine hydrochloride ophthalmic solution (Bausch & Lomb, Tampa, FL) followed by a drop of 5% betadine solution (Alcon, Fort Worth, TX) was administered to the operative eye and allowed to dry for 5 min. MMC (4 mg mL^−1^, 50 µL) was injected into the superotemporal subconjunctival space of rabbits receiving PCS and CPS implants using a 30-guage needle. After placement of an 8–0 silk, stay suture (Ethicon, Somerville, NJ) in the superotemporal limbus, a two-clock hour, fornix-based, conjunctival peritomy was created, and the conjunctiva was dissected posteriorly. A 25 G needle (Fisher Scientific, Waltham, MA) was used to create a 2 mm long scleral tunnel prior to entering the anterior chamber. The shunt was gently guided through this tunnel. Once the shunt was in position, the template wire was removed and a 10–0 nylon cross-stitch (Ethicon) was placed at the site of tube entry to prevent fluid leakage around the tube. Balanced saline solution (BSS, Alcon) was irrigated in the anterior chamber using a 30 G needle (Fisher Scientific) to validate shunt patency. Conjunctiva was approximated to the limbus using two 10–0 nylon sutures and, again, BSS was irrigated into the anterior chamber to ensure that a water-tight bleb had formed. The stay suture was removed and topical antibiotic ointment was administered to the eye. In this manner, 3 open- and closed-lumen (containing the template wire) PS, 3 open-lumen PCS, and 4 open-lumen CPS were implanted. All shunts were 6 mm in length. IOP was measured and calibrated as previously reported^48^. Baseline IOP reported in Table S1 was measured in non-operated rabbits for open- and closed-lumen PS, and pre-operatively in the operated eye for PCS and CPS. IOP was measured post-operatively and at 1, 4, 7, 11, 14, 21, and 27 days for eyes containing PS and PCS using a TonoVet (iCare, Vantaa, Finland) rebound tonometer in awake, restrained rabbits without topical anesthesia as described previously^49^. Eyes containing a CPS implant were evaluated on day 1 post-operatively after which the rabbits were euthanized. A modified Moorfields bleb grading system was used to assess bleb size, height, and vascularity in all eyes and eyes were clinically evaluated for inflammation, infection, and anterior chamber pathology^50^. Shunt patency was evaluated weekly via irrigation of fluorescein (Sigma Aldrich) into the anterior chamber. 0.1% fluorescein (< 0.05 mL) was injected manually into the anterior chamber and care was taken not to elevate IOP above 25 mmHg. Fluorescein venting to the subconjunctival space was graded as either a positive (fluorescein visualized in the subconjunctival space through the shunt lumen), negative (no fluorescein), or leak (fluorescein in the subconjunctival space but not through the shunt lumen). Prior to fluorescein irrigation procedures, eyes were examined under an operative microscope for anterior chamber inflammation or fibrin formation, anterior chamber neovascularization, cornea neovascularization, cataract formation, and other signs of anterior chamber or surface pathology.

### Assessment of in vivo biocompatibility

Open- and closed-lumen PS (n = 3, each), and PCS (n = 3) were implanted into NZW rabbits via an *ab externo* procedure, as described. After monitoring for 27 days, rabbits were euthanized and eyes enucleated, fixed in formalin, embedded in paraffin, cross-sectioned, and stained with H&E and Masson’s trichrome for histological evaluation.

### Statistical analysis

Pressure and IOP measurements are presented as mean ± SE for specific time points and as mean ± SD for averages of all IOPs throughout the duration of the experiment. Shunt dimensions are also presented as mean ± SD. IOP data were analyzed using a Student’s t-test, and differences between parameters were considered statistically significant for p values < 0.05. Statistical significance of post-operative day 1 IOP values for control, PCS, and CPS eyes was determined via one-way ANOVA followed by Tukey test.

## Supplementary information

Supplementary information

## Data Availability

All data generated or analysed during this study are included in this published article and its Supplementary Information files.
