# Supplementary material for: Nano-structured glaucoma drainage implant safely and significantly reduces intraocular pressure in rabbits via post-operative outflow modulation
Source: Sci Rep. 2020 Jul 31;10:12911. doi: 10.1038/s41598-020-69687-4 (PMC7395089; doi:10.1038/s41598-020-69687-4)
Supplement: Supplementary file 1 — Supplementary information [file 41598_2020_69687_MOESM1_ESM.pdf]

Nano-structured glaucoma drainage implant safely and significantly reduces intraocular pressure in rabbits via post-operative outflow modulation

Kunal S. Parikh[1,2,3,4+], Aditya Josyula[1,5+], Revaz Omiadze[1,4], Ju Young Ahn[1,2], Youlim Ha[1,5], Laura Ensign[1,2,4,5,6], Justin Hanes[1,2,4,5,6,8], Ian Pitha[1,4,7*]

[1] Center for Nanomedicine, Johns Hopkins University School of Medicine, Baltimore, MD 21231, USA.
[2] Department of Biomedical Engineering, Johns Hopkins University School of Medicine, Baltimore, MD 21205, USA.
[3] Center for Bioengineering Innovation & Design, Johns Hopkins University, Baltimore, MD 21218, USA.
[4] Department of Ophthalmology, Wilmer Eye Institute, Johns Hopkins University School of Medicine, Baltimore, MD 21231, USA.
[5] Department of Chemical and Biomolecular Engineering, Johns Hopkins University, Baltimore, MD 21218, USA.
[6] Department of Pharmacology and Molecular Sciences, Johns Hopkins University School of Medicine, Baltimore, MD 21205, USA
[7] Glaucoma Center of Excellence, Wilmer Eye Institute, Johns Hopkins University, Baltimore, MD 21287, USA.
[8] Departments of Environmental Health Sciences, Oncology, and Neurosurgery, Johns Hopkins University School of Medicine, Baltimore, MD 21231, USA.

[*]ipitha1@jhmi.edu.

[+]these authors contributed equally to this work

Supplementary Material

Table S1. Baseline and post-operative IOP measurements in rabbits receiving PS, PCS, and CPS implants. Note that p values for comparison of baseline and post-operative IOP values are not applicable for Closed PS and Open PS due to lack of variance in data points.

Shunt	Mean Baseline IOP (mmHg)	Mean Post-operative IOP (day)	IOP Reduction (%)	P-value
**Closed PS**	17.1	14.9 (27)	2.2 (12.9)	N/A
**Open PS**	17.1	11.6 (27)	5.5 (32.2)	N/A
**PCS**	18.6	8.3 (27)	10.3 (54.2)	$p < 0.05$
**CPS**	13.3	5.3 (1)	8 (61.5)	$p < 0.001$

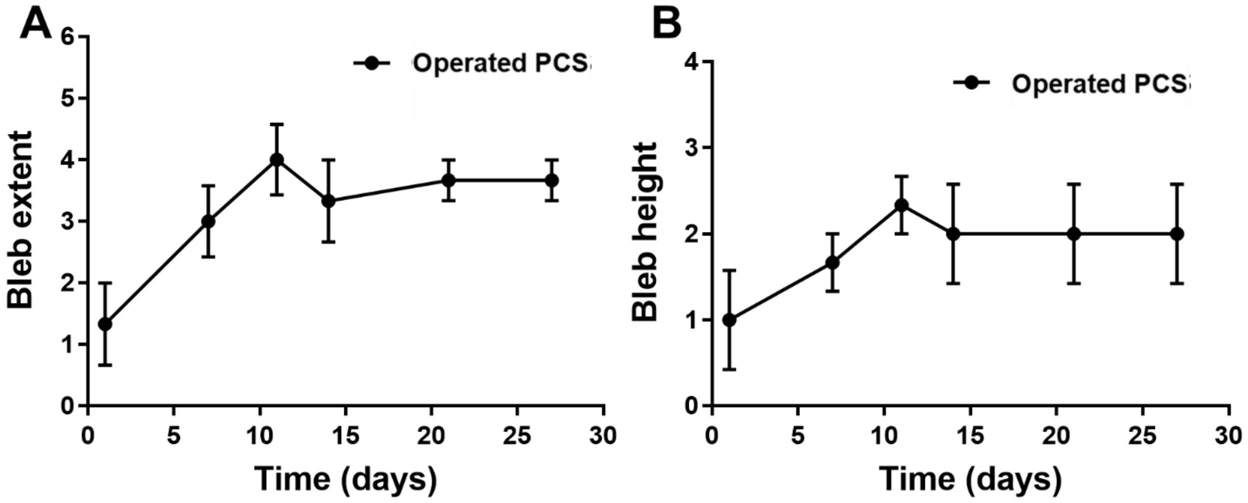

Figure S1. Bleb grading following implantation of PCS.

A modified Moorfields bleb grading system was used to assess bleb size and height post-operatively in rabbit eyes containing PCS. Eyes containing PCS demonstrate a gradual increase in magnitude of bleb extent (A) and elevation (B) and retain bleb morphology throughout the postoperative period.

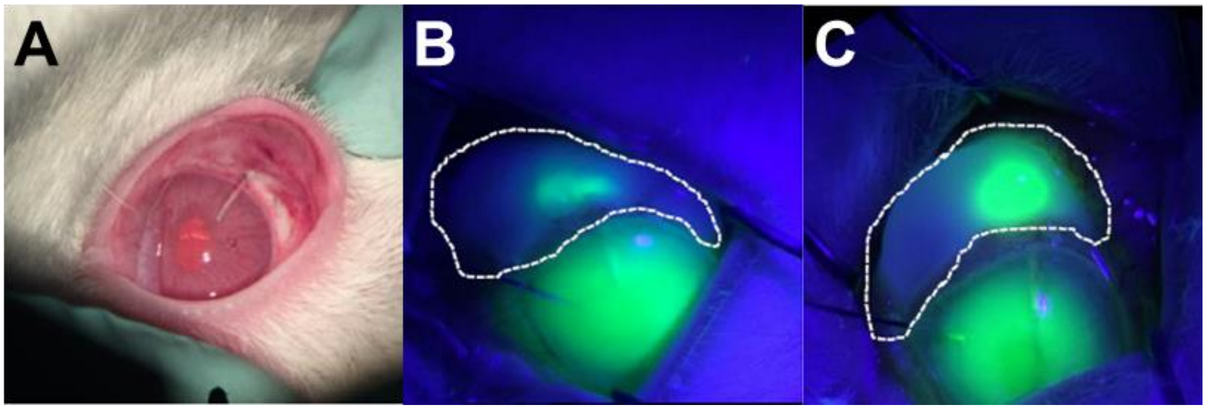

Figure S2. PCS Implantation and patency.

Representative images of (A) PCS implantation on postoperative day 1 with the proximal end in the anterior chamber and the distal end in the subconjunctival space, and UV light visualization following anterior chamber irrigation with fluorescein sodium at post-operative days (B) 11 and (C) 27. Dye diffuses into the subconjunctival space at the distal end of the stent demonstrating stent patency throughout the duration of implantation.

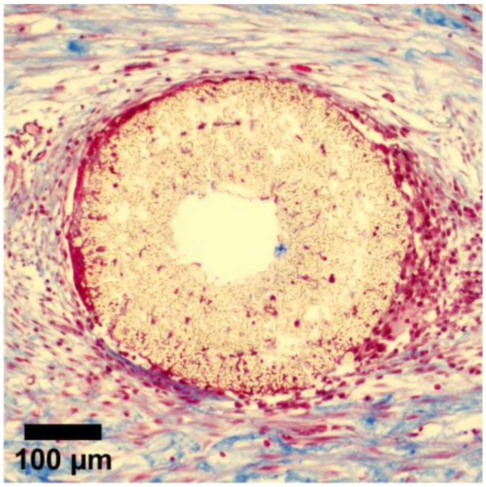

Figure S3. PCS biocompatibility. Representative image of tissue surrounding an implanted, open PCS following Masson's trichrome staining.
